# Epigenetic responses of hare barley (*Hordeum murinum* subsp. *leporinum*) to climate change: an experimental, trait-based approach

**DOI:** 10.1038/s41437-021-00415-y

**Published:** 2021-02-19

**Authors:** Víctor Chano, Tania Domínguez-Flores, Maria Dolores Hidalgo-Galvez, Jesús Rodríguez-Calcerrada, Ignacio Manuel Pérez-Ramos

**Affiliations:** 1 Research Group “Sistemas Forestales Mediterráneos”, Instituto de Recursos Naturales y Agrobiología de Sevilla. Dpto, Biogeoquímica, Ecología Vegetal y Microbiana, Consejo Superior de Investigaciones Científicas, Av. Reina Mercedes 10, 41012 Sevilla, Spain; 2 Research Group “Sistemas Naturales e Historia Forestal”, ETSI Montes, Forestal y del Medio Natural. Dpto, Sistemas y Recursos Naturales, Universidad Politécnica de Madrid, Ciudad Universitaria s/n, 28040 Madrid, Spain; 3 Present Address: Department of Forest Genetics and Forest Tree Breeding, University of Göttingen, Büsgenweg 2, 37077 Göttingen, Germany

**Keywords:** Climate-change ecology, Molecular ecology

## Abstract

The impact of reduced rainfall and increased temperatures forecasted by climate change models on plant communities will depend on the capacity of plant species to acclimate and adapt to new environmental conditions. The acclimation process is mainly driven by epigenetic regulation, including structural and chemical modifications on the genome that do not affect the nucleotide sequence. In plants, one of the best-known epigenetic mechanisms is cytosine-methylation. We evaluated the impact of 30% reduced rainfall (hereafter “drought” treatment; D), 3 °C increased air temperature (“warming”; W), and the combination of D and W (WD) on the phenotypic and epigenetic variability of *Hordeum murinum* subsp. *leporinum* L., a grass species of high relevance in Mediterranean agroforestry systems. A full factorial experiment was set up in a savannah-like ecosystem located in southwestern Spain. *H. murinum* exhibited a large phenotypic plasticity in response to climatic conditions. Plants subjected to warmer conditions (i.e., W and WD treatments) flowered earlier, and those subjected to combined stress (WD) showed a higher investment in leaf area per unit of leaf mass (i.e., higher SLA) and produced heavier seeds. Our results also indicated that both the level and patterns of methylation varied substantially with the climatic treatments, with the combination of D and W inducing a clearly different epigenetic response compared to that promoted by D and W separately. The main conclusion achieved in this work suggests a potential role of epigenetic regulation of gene expression for the maintenance of homoeostasis and functional stability under future climate change scenarios.

## Introduction

There is a growing interest in understanding how plants will adapt to the projected changes in climate. Plant species respond to environmental changes by means of multiple morphological and physiological adjustments that serve to alleviate stress levels and to increase the uptake of limiting resources (Nicotra et al. 2010; Freschet et al. 2018; Pérez-Ramos et al. 2019). For example, plants subjected to greater water/nutrient limitations usually exhibit a suite of trait values associated to efficient resource conservation use (e.g., plants with small-sized and high-density leaves) (Chapin et al. 1993; Wright et al. 2004), which increases competitive abilities under resource-limiting conditions. This strategy contrasts with that displayed by plants inhabiting moist and fertile sites, with opposite trait values related to a rapid return on investment. Moreover, under non-limiting conditions of soil water and nutrient availability, an increase in air temperature could result in thinner leaves with higher specific leaf area that favour plant carbon uptake and growth (Poorter et al. 2009; Lamaoui et al. 2018). Plants can also modify their reproductive output and phenology under different environmental scenarios of temperature and resource limitation (Valencia et al. 2016; Pérez-Ramos et al. 2020).

The species’ ability to vary its phenotypic expression across environments is mainly driven by genetic variability, which is in turn originated by sexual reproduction and random mutations (Ewens 2013). In addition, there are evidences that epigenetic processes (i.e., structural and chemical modifications on the genome that do not affect the nucleotide sequence) can promote fast and reversible phenotypic variations in response to environmental changes (Bossdorf et al. 2008). These epigenetic mechanisms act in a switch mode, activating/deactivating gene transcription in three different ways: (i) DNA methylation by the covalent binding of methyl groups to cytosine nucleotides (5 mC), (ii) regulation of DNA accessibility by histone modification and (iii) post-transcriptional regulation by non-coding RNAs activity such as microRNAs (Bossdorf et al. 2008). In plants, DNA methylation has been found to silence transposable elements (TEs) and gene expression (Bartels et al. 2018), with promoter methylated genes having lower transcription levels (Zhang et al. 2006; Li et al. 2012). Recent studies have argued that epigenetic mechanisms could play a relevant role in microevolution under challenging environmental scenarios, such as those promoted by climate change (e.g., Kronholm et al. 2017; Jeremias et al. 2018; Münzbergová et al. 2019). In fact, although it is broadly assumed that species with high genetic variability have higher adaptation potential for a larger variety of environmental conditions than species with low genetic diversity (Anderson et al. 2011), recent studies (e.g., Zhang et al. 2016) have demonstrated the importance of epigenetic diversity for environmental adaptation in plant species with limited genetic diversity. Notwithstanding, there are still many gaps on the role of epigenetics as a driver of this source of phenotypic variability across contrasted environments.

Several studies have reported the influence of epigenetic responses to different environmental conditions on some developmental processes. For instance, it has been demonstrated that phenotypic variations in *Viola cazorlensis* and allopolyploid orchids in response to climate are mediated by both genetic and epigenetic processes (Herrera and Bazaga 2010; Paun et al. 2010). Other works have also focused on the epigenetic responses to different sources of stress, both biotic, such as herbivory, disease or plant competition for nutrients (Conrath et al. 2002; Tani et al. 2005; Verhoeven et al. 2010; Jaskiewicz et al. 2011), and abiotic, such as salinity, mechanical stress, heat and drought (Herrera et al. 2012; Latzel et al. 2013; Liu et al. 2015; Alsdurf et al. 2016; Banerjee and Roychoudhury 2017). Most of these studies have been conducted under controlled conditions or in natural populations differing in a limited (one or two) number of factors. However, in nature, plants are exposed to a variety of constraints, which constitute a multidimensional space where many factors act simultaneously and interactively (Ibáñez and Schupp 2001; Gómez 2004). Since the effect of one stress factor on plant performance may be exacerbated or mitigated by another (Mitchell et al. 2015), the impact of a combination of various stress factors may differ from the sum of the impacts caused by each factor applied individually. As a result, combined stress factors may enhance phenotypic variation and plant fitness despite the presumably higher costs of plasticity (Lampei 2019). Therefore, an accurate prediction of climate change projections on plant phenotypic variability requires the consideration of potential additive and interactive effects of different abiotic factors.

The role of methylation or demethylation processes in response to stress has not been fully elucidated and seems to be dynamic in time (Liu et al. 2018). For instance, several alleles were identified and related to a plastic response to climate variation in natural populations of *Arabidopsis thaliana*, with diverse genome-wide methylation patterns associated to seasonality (Shen et al. 2014). Wang et al. (2011) detected different patterns of genome-wide methylation/demethylation in rice induced by water limitation, with plants exhibiting 70% reversibility to the original status after drought cessation. Another study also detected drought-induced responses in rice, with hyper- and hypo-methylation being related to susceptibility and tolerance to water deficit, respectively (Gayacharan 2013). On the other hand, DNA-methylation processes have been widely studied in response to heat stress, especially through the activity of methyltransferases (see review by Liu et al. 2015). All these studies suggest that methylation patterns in response to stress conditions depend on the source of stress, as well as on the genotype, the tissue and the ontogeny (Bonasio et al. 2010; Tan 2010; Wang et al. 2011; Mastan et al. 2012), and that these factors will likely encompass a wide range of responsive genes.

In the present study we analysed the two main sources of genotypic variability (i.e., genetic and epigenetic) of an annual grass species dominant in Mediterranean savannah-like ecosystems, *Hordeum murinum* subsp. *leporinum* L. (hare barley, hereafter), with great relevance in pasture dynamics due to its fast growth and high palatability for livestock (Hulting and Haavisto 2013). This species is widely distributed in Europe and Middle East and has been declared as moderately invasive in California by the California Plant Invasive Council (Supplementary Data Fig. S1). Plants were experimentally subjected to increased temperature, decreased rainfall, and the combination of both factors. We also examined the phenotypic responses of this species to the different climatic treatments by measuring eight functional traits related to plant morphology, phenology and reproductive ability. The effort of combining epigenetic analysis under contrasting climatic conditions with plant phenotypic characterisation using attributes related with the three leading dimensions of ecological variation (i.e., plant economics, light interception, and reproductive ability) (Westoby 1998; Laughlin et al. 2010; de la Riva et al. 2016) aims to increase our understanding on the role of epigenetic regulation as a driver of plant acclimation to ongoing climate changes. In fact, global change models predict an increase in temperature and more severe and recurrent drought periods for the next decades in many temperate and Mediterranean ecosystems (Lindner et al. 2010). In this context, savannah-like ecosystems in the Iberian Peninsula (known as *dehesa* in Spain and *montados* in Portugal) may be particularly sensitive to increased temperatures and decreased rainfall (Olea and San Miguel 2006). These environmental changes could potentially jeopardise the weak equilibrium of their plant communities, which already suffer the inclement conditions of low soil water availability and high temperatures in summer. We hypothesise that hare barley will display different epigenetic and functional responses to the different environmental scenarios, with the combination of increased temperature and reduced rainfall resulting in a differential response compared with the responses resulting from standalone climatic treatments. More specifically, we hypothesise that plants subjected to warmer conditions will exhibit earlier reproductive phenology and higher plant height, and plants subjected to decreased rainfall will show trait values associated to higher efficient resource conservation (i.e., small-sized and high-density leaves).

## Material and methods

### Study site and experimental conditions

The study was carried out in *“La Morra”* (X:340933, Y:4246190), a *dehesa* located in southwestern Spain (*Los Pedroches* valley, Córdoba). Climate is Mediterranean, with cool wet winters and hot dry summers. Mean annual rainfall is 428.2 mm, and mean annual temperature is 14.6 °C (with a mean monthly maximum of 34.7 °C in July and a mean monthly minimum of 0.7 °C in January; mean values for the 2008–2017 period). Vegetation is characterised by a dense cover of herbaceous species (≥ 65% relative plant cover; most of them annuals), which coexist with scattered oaks (mostly *Quercus ilex*; ~20%) and shrub species (≤7.5%, mostly *Crataegus monogyna* and *Retama sphaerocarpa*).

In September 2016, before the beginning of the rainy season, a factorial experiment was designed with four climatic treatments: warming (W, hereafter), drought (D), warming and drought (WD), plus a control treatment (C) of plants subjected to natural conditions. The four treatments were replicated in six plots of 4 × 6 m, with a minimum distance of 20 m from each other, and fenced with a metallic structure to avoid livestock access (Supplementary Data Fig. S2). The drought treatment was created by placing 6 methacrylate gutters, 0.14-m wide each, inclined 20° over half of every plot (2.5 × 2.5 × 1.5 m). These rainout shelters reduced the total amount of rainfall reaching the soil surface by around 33%. To recreate warming conditions, hexagonal open-top chambers (OTC) were used (see Marion et al. 1997). They were built with methacrylate sheets without UV-Filter to avoid modifying the light spectrum, with sloping sides of 40 × 50 × 32 cm, and wavelength transmission between 280 and 750 nm. Previous studies indicated that OTCs increase air temperature by 1–3 °C relative to the external environment without altering light transmission within the chambers (Dabros and Fyles 2010; Aragón-Gastélum et al. 2014). In each plot, two OTCs were placed under the rainout shelters in order to evaluate the impact of temperature increase and rainfall exclusion simultaneously (WD treatment) and two outside (W treatment). These permanent structures of rainfall exclusion and temperature rise were chosen due to their successful use in past studies on climate change simulations (e.g., Delgado-Baquerizo et al. 2013; Maestre et al. 2013).

Soil volumetric water content was quantified using a capacitance soil moisture probe (Delta T Devices). In half of the experimental plots, one 40-cm long tube was inserted into the soil per climatic treatment, and measurements of soil humidity were taken periodically from November to April. Soil surface air temperature was quantified hourly by means of HOBO-type sensors (Alpha Omega Electronics) in half of the experimental plots (three replicates per climatic treatment). As expected, air temperature was higher in the experimental units located within the OTCs (i.e., W and WD treatments), whereas soil humidity decreased in the units located beneath the rainout shelters (i.e., D and WD treatments; see Supplementary Data Fig. S3).

### Plant phenotypic measurements

In 24 experimental units (i.e., 6 plots × 4 climatic treatments), four 21 × 21 cm PVC quadrats (divided in turn into nine squares of 7 × 7 cm) were placed at the peak of maximum vegetative growth (i.e., in the mid-spring of 2017 and 2018) to determine species abundance and composition. Species frequencies were calculated from the number of squares where each species was present. The relative frequency of hare barley and its temporal evolution from 2017 to 2018 was further compared to those of the co-dominant species *Avena barbata, Crepis capillaris, Erodium moschetum, Geranium dissectum* and *Sinapis alba*. These five species were selected because they were among the most abundant in the different experimental units and their relative frequencies varied widely from the first to the second sampling year.

In April 2017, at peak biomass, 10–30 individuals of hare barley per climatic treatment were randomly selected to measure one whole-plant trait (plant height), one reproductive trait (seed mass) and three morphological above-ground traits: leaf size, specific leaf area (SLA; leaf area per unit of leaf dry mass), and leaf dry matter content (LDMC; dry mass per unit of water-saturated fresh mass). All these traits were measured at the level of treatment instead of at the plot level due to the low number of individuals present in some plots. Thus, plant height was measured in 30 plants per climatic treatment using a calliper. Leaf size, SLA and LDMC were quantified in ten plants per climatic treatment, following the protocols described by Garnier et al. (2001). Leaf size was quantified using an image analysis programme (Image Pro-plus 4.5; Media Cybernetic Inc., Rockville, MD, USA). Seed mass was quantified by weighing all the seeds contained in one infrutescence per plant, in at least 10 plants per climatic treatment; seeds were previously oven-dried at 60 °C for 48 h to obtain their dry weight. Additionally, reproductive phenology at the population level was monitored once a week over the whole period of flowering of the species (from mid-February to late June 2017). In each census, we counted the number of flowers of hare barley using a semi-quantitative scale (from 0 to 5). We registered the specific dates in which the onset, peak and cessation of flowering took place in those experimental units where hare barley was present. The onset of flowering was defined as the date when the first flower was observed in at least one individual, whereas the cessation of flowering was defined as the date when the last flower wilted. The difference between the onset and the cessation of flowering was used to calculate the duration of flowering. The peak of flowering was defined as the date when the population reached its maximal number of flowers. All these traits were selected for covering the three dimensions of ecological variation among plants (i.e., plant economics, light interception, and reproductive ability) (Westoby 1998; Laughlin et al. 2010; de la Riva et al. 2016), given their utility in studies of abiotic stress and functional ecology (Wright et al. 2004; Pérez-Ramos et al. 2017).

### Sample collection and DNA extraction

In April 2017, 30 individuals of hare barley per climatic treatment (five per plot) were collected, discarding the inflorescences, and immediately frozen in liquid nitrogen to avoid DNA methylation due to sampling. In the lab, frozen samples were ground, and 100 mg of tissue powder was used for DNA extraction using the CTAB method (Doyle and Doyle 1987). The isolated DNAs were quantified with a NanoDrop 2000 UV (Thermo scientific).

### AFLP analysis

Twenty samples from the C treatment distributed among the 6 experimental plots were used for the analysis of genetic variation between plots by using Amplified Fragment Length Polymorphism (AFLP). This tool is based on the analysis of DNA markers resulting from the fragmentation of DNA using EcoRI and MseI restriction enzymes (Vos et al. 1995), which cut a specific sequence of nucleotides in the DNA. For each individual sample reaction, 500 ng of total DNA were digested at 37 °C for 3 h and 100 rpm using 5 U of EcorRI (New England Biolabs) and 5 U of MseI (New England Biolabs) in a final volume of 25 µl. The DNA fragments were then ligated to double-stranded EcoRI and MseI adaptors (Supplementary Data Table S1), in a final volume of 30 µl, using 1 U of T4 DNA ligase (New England Biolabs) at 37 °C during 6 h and 100 rpm, and then overnight at 4 °C. In order to subset the number of amplified fragments, a 20 µl pre-selective PCR reaction was carried out using the product of the ligation reaction (for primers information see Supplementary Data Table S1). PCR conditions were as follows: denaturing at 94 °C for 5 min, 28 cycles of 94 °C for 30 s, 60 °C for 60 s, 72 °C for 60 s, and a final elongation step at 72 °C for 10 min. The product of the first pre-selective PCR was used as template for a subsequent 10 µl selective PCR reaction, with the following conditions: 94 °C for 5 min, 12 cycles of 94 °C for 30 s, 65–56 °C for 30 s (decreasing 0.7 °C each cycle), 72 °C for 6 s, 23 cycles of 94 °C for 30 s, 56 °C for 30 s, 72 °C for 60 s, and a final elongation step of 72 °C for 5 min. This selective PCR was performed using 4 primer combinations (Supplementary Data Table S1). Resulting DNA fragments were analysed through the electrophoretic system 4300 DNA Analyser System (LiCOR Bioscience), along with a 50–1500 bp Size Standard (LiCOR Bioscience). DNA fragments between 100 bp and 500 bp were included into the analysis. According to the presence/absence of bands for specific positions, referred to as *locus*, a Boolean datamatrix coded by 0 (absence) and 1 (presence) was constructed.

### MSAP analysis

DNA methylation patterns were identified by Methyl-Sensitive Amplified Polymorphism (MSAP) for the 120 individuals of hare barley collected. This methodology, as AFLP, is based on DNA markers from DNA digestion, but uses EcoRI/HpaII and EcoRI/MspI couples of restriction enzymes in two parallel reactions (Reyna-López et al. 1997). The main difference between AFLP and MSAP is that with MSAP the isoschizomers HpaII and MspI cut the target depending on the methylation state of the cytosines present in the sequence. As for AFLP, the protocol involved 500 ng of total DNA for each reaction, and the same conditions during digestion, adaptors ligation, and PCR. Moreover, a selective PCR was carried out using 12 primer combinations for each restriction enzyme couple (Supplementary Data Table S1) and the resulting DNA fragments between 100 bp and 500 bp were used to construct the Boolean datamatrix.

### Detection of global DNA methylation levels

A global 5-methylcytosine analysis (5-mC DNA ELISA Kit, ZYMO) was used to measure the global DNA methylation levels in hare barley in response to climatic treatments. DNA from seven individuals per treatment randomly selected was analysed. The optical density at 405 nm was determined after 45 min using an Absorbance Microplate Reader ELx808^TM^ (Bio-Tek^®^ Instruments, Inc., USA). The global DNA methylation levels were expressed in percentage of DNA as the mean of three technical replicates according to the manufacturer instructions.

### Statistical and bioinformatic analysis

The effects of the experimental climatic treatments (W, D and WD) were tested on the eight phenotypic variables related with plant morphology (plant height, leaf size, SLA and LDMC), reproductive output (seed mass) and plant phenology (flowering onset, peak and duration), as well as on the global 5-methylcytosine levels. First, the distribution of data sets was assessed in R by means of the Shapiro–Wilk test, and secondly homoscedasticity was analysed by using Bartlett or Levene test depending on whether data was normally distributed or not. For homoscedastic data, differences between treatments were tested by means of ANOVA (Analysis of Variance) or Kruskal–Wallis test, depending on whether data was normally distributed or not, respectively, while for heteroscedastic data a Welch’s ANOVA was used. Finally, post-hoc analyses were performed when a dependent variable differed between climatic treatments, using Tukey’s, Dunn–Bonferroni, and Games-Howell multiple comparisons test depending on the assessment used (ANOVA, Kruskal–Wallis or Welch-ANOVA, respectively).

The *msap* v1.1.9 package (Pérez-Figueroa 2013) developed in R (R Core Team 2013) was used for the analysis of both MSAP and AFLP matrices. For MSAP, this package detects the activity of each restriction enzyme and classifies each *locus* depending on the methylation state of the cytosines present in the target sequence (Supplementary Data Table S2). Thus, the *loci* were classified as Methylation-Susceptible Loci (MSL), which are used to assess epigenetic variation, or Non-Methylated Loci (NML), used as a proxy to assess genetic variation (Watson et al. 2018). Variations among MSL were also calculated with the Shannon’s diversity index (*S*), and epigenetic variations among plots and climatic treatments were also explored by principal coordinate analyses (PCoA) based on Euclidean distance matrix, implemented in the *msap* package. Furthermore, an analysis of the molecular variance (AMOVA) was used for the estimation of variance components and the *Phi*-statistic (analogue to the F-statistic for binary data) to reflect genetic and epigenetic diversity (Excoffier et al. 1992). In order to determine those *loci* showing non-randomly distributed methylation patterns between treatments (h for hemimethylation, i for methylation of inner cytosines, u for non-methylation, and f for uninformative state, which may be due to full methylation or changes in the nucleotide sequence of the target), a *locus*-by-*locus Chi*-squared test was performed using the MSL, following the reproducible example script for R found in Watson et al. (2018). To control the false discovery rate (FDR), a BH multitest adjustment was adopted (Benjamini and Hochberg 1995), and those MSL with *p-value* < 0.001 were considered as significantly differentiated. The relationships between significantly differentiated loci were calculated with the Gower’s Coefficient of Similarity (Gower 1971), and the *Complex Heatmap* package available for Bioconductor in R (Gu et al. 2016) was used to cluster and show the resulting matrix as a heatmap.

Genetic diversity among the six experimental plots was assessed by means of AFLP technique using twenty samples from the C treatment. As stated before, this tool is based on the fragmentation of DNA using EcoRI and MseI restriction enzymes (Vos et al. 1995), which cut a specific sequence of nucleotides in the DNA. The statistical analysis was performed using the *msap* package but configured to analyse AFLP data by using the logical value meth = FALSE. As done for epigenetic analysis, genetic variations among plots were also explored by PCoA and the AMOVA. As mentioned above, genetic variation among treatments was also assessed by using NML, whose banding patterns depend on variations at restriction sequence. This approach is appropriate when a high number of NML is detected in a representative sample size (Pérez-Figueroa 2013; Watson et al. 2018).

## Results

### Plant phenotypic measurements

**Fig. 1:**
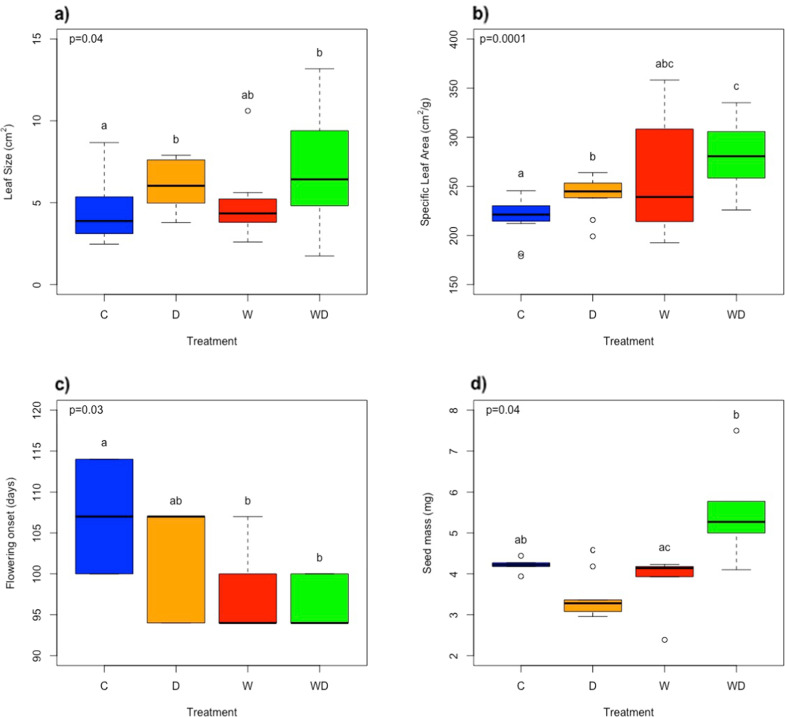
Changes in some key phenotypic traits as a function of the climatic treatment. **a** Leaf size; **b** Specific Leaf Area; **c** Flowering onset and; **d** Seed mass. p *p*-value from overall effect test. Different letters above bars denote significantly different groups after post-hoc test (FDR adj. *p* value < 0.05).

Of the eight phenotypic traits considered in this study, four exhibited significant differences among treatments (*p* value < 0.05): Leaf Size (LS), Specific Leaf Area (SLA), Flowering Onset and Seed Mass (Supplementary Data Table S3). Among the three foliar traits, just LDMC did not significantly differ among treatments. Post-hoc comparisons indicated that plants subjected to drought (D and WD) had significantly higher LS and SLA than C plants (Figs. 1a, b). However, differences in LS between D and WD were not significant at *p* value < 0.05, while differences in SLA between these treatments were statistically significant (Supplementary Data Table S3). Plants exposed to warming without drought (W) also tended to have higher SLA than C plants, but differences were not significant at *p* value 0.05. Regarding reproductive phenology, warming accelerated the onset of flowering in about ten days (both in W and WD treatments; Fig. 1c) compared with those plants growing under control conditions. Finally, significant differences in seed mass were found among treatments, with WD plants producing seeds of higher weight, especially when compared to plants subjected to drought (Fig. 1d).

### Intrapopulation genetic diversity

**Fig. 2:**
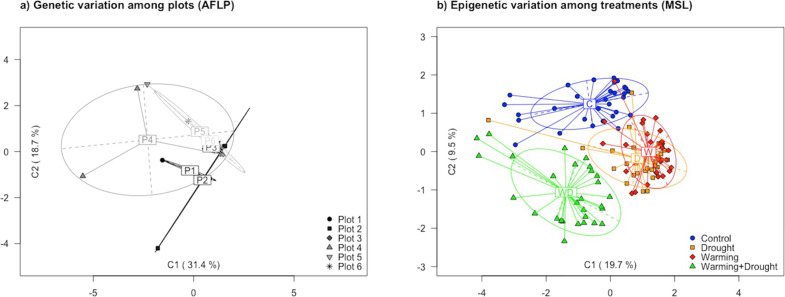
Principal Coordinate Analyses. **a** Results from PCoA to analyse the genetic variation of hare barley among six experimental plots. **b** Results from PCoA to analyse the epigenetic variation in response to climatic treatments. Labels indicate the centroids, and ellipses show the dispersion associated to each plot (**a**) or climatic treatment (**b**).

The AFLP technique was used for genotyping 20 individuals from the C treatment. The number of *loci* produced by four selective primer combinations was 26, 26, 12 and 12 (for E34-M53, E35-M53, E34-M56 and E35-M56, respectively). The percentage of polymorphic bands from the total of 75 loci analysed was 92%. Very low genetic divergence was found within this population, and differences among plots were not significant (*Φ*_ST_ value of 0.0323; *p* value = 0.278; Table 1). The AMOVA test revealed that genetic differences mostly occurred within plots (almost 99% of the variation), whereas just 1% was observed between plots. A pairwise AMOVA also confirmed the lack of separation between plots (Supplementary Table S4). In addition, the PCoA did not show a clear separation of plants, and plots were overlapped with each other (Fig. 2a). The total variance explained reached 50.1%, with the first component of the PCoA explaining 31.4%.

**Table 1:** Analysis of molecular variance (AMOVA) for genetic (based on AFLP data) and epigenetic (based on MSAP data) diversity.

Source of variation	d.f.	SS	MSS	Variance	Percentage of variation (%)	*Φ*_ST_ (*p* value)
*AFLP analysis for genetic variability*						
*Among plots*	5	60.96	12.19	0.3667	3.23	0.0323 (>0.05)^ns^
Within plots	14	153.7	10.98	10.98	96.77	
Total	19	214.6	11.3			
*MSAP analysis for epigenetic variability*						
*Among treatments*	3	322.1	107.4	3.294	27.81	0.2781 (<0.0001)
*Within treatments*	116	991.9	8.551	8.551	72.19	
*Total*	119	1314	11.04			

*d.f*. degrees of freedom, *SS* sum of squares, *MSS* mean sums of squares, ^ns^ not significant.

### Epigenetic variability

**Fig. 3:**
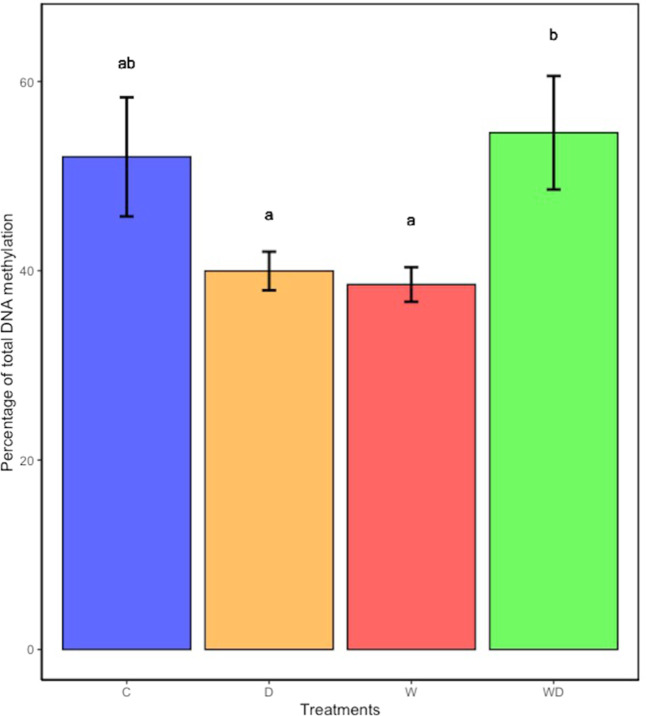
Global DNA methylation (mean of percentage ± SE) of methylated cytosines found for each climatic treatment. Different letters above bars denote significantly different groups after post-hoc test (FDR adj. *p* value < 0.1).

Climatic treatments induced significant differences among plants in both the percentage of cytosines methylated and the pattern of methylation. On the one hand, the quantification of 5-mC by ELISA (Enzyme-Linked Immunosorbent Assay; Zymo) yielded percentages of methylated cytosines ranging from 30.8 to 76.4%. As shown in Fig. 3, the higher percentages of methylated cytosines were found both in C (mean = 52.03 %) and WD (mean = 55.08 %), compared to D (mean = 39.89%) and W (mean = 38.55%). Welch’s ANOVA (used for normally distributed heteroscedastic data) resulted in a marginal significance (*p* value = 0.06), and post-hoc Games–Howell analysis for pair-treatments comparisons revealed significant differences between WD plants and those subjected to standalone stressors (*p value* < 0.1).

For the determination of epigenetic divergences among treatments, a total of 354 loci in 120 individuals (42,480 fragments) were analysed with the *msap* package using 12 primer combinations. From this total number of loci, 343 were classified as susceptible to be methylated (MSL), and 62 of them were polymorphic (18%). The Shannon diversity Index (*S*) for MSL was *S* = 0.51 ± 0.14 (mean ± SD). As shown in Table 1, differences in DNA methylation between treatments were highly significant, with a *Φ*_ST_ value from AMOVA for epigenetic variation among treatments of 0.2781 (*p* value < 0.0001). The remaining 11 loci were considered as NML, of which five were polymorphic (45%). However, this approach is not appropriate for inferring genetic variation when very low numbers of NML are detected.

In Fig. 2b, a PCoA revealed multi-*locus* epigenetic differentiation between climatic treatments. Along the first coordinate, which explained 19.7% of the total variance, a clear separation was observed between climatic treatments, with W and D appearing in one side and C and WD in the other side. Moreover, the C and WD treatments were separated along the second coordinate, explaining almost 9.5% of total variance. In addition, pairwise AMOVAs (Table 2) reflected the differentiation among treatments for MSL diversity (epigenetic); epigenetic divergence between treatments were significant for all pairwise comparisons.

**Table 2:** Results from pairwise analyses of molecular variance (AMOVA) between pairs of climatic treatments.

	C	D	W	WD
C	–			
D	0.2818*	–		
W	0.2856*	0.1882*	–	
WD	0.2322*	0.3070*	0.3464*	–

Values correspond to *Phi*-statistic based on MSL *loci*.

*C* control; *D* drought; *W* warming; *WD* warming + drought; *p value < 0.0001.

Plants subjected to different climatic treatments exhibited distinct methylation patterns. Table 3 shows the frequency of the different methylation states detected in the different climatic treatments. Differences among treatments were small, although some remarkable differences were observed: (i) the uninformative methylation state of MSAP *loci* showed lower frequencies in D and W than in C and WD treatments; (ii) the unmethylated state in D and W was higher than in C and WD; (iii) the hemimethylation state was more frequent in response to all climatic treatments than in C, being higher in D and W than in WD; and (iv) methylation of inner cytosines was less frequent in response to the combined treatment WD.

**Table 3:** Frequency (%) of methylation states at the target sequence for each climatic treatment after multi-*locus* analysis.

DNA methylation state	Control	Drought	Warming	Warming + Drought
*Unmethylated*	8.9	12.5	12.9	8.5
*Hem*imethylated	25.2	31.1	31.3	28.4
Methylation of internal cytosine	42.7	40.7	41.6	37.0
Uninformative	24.2	15.7	14.2	26.1

**Fig. 4:**
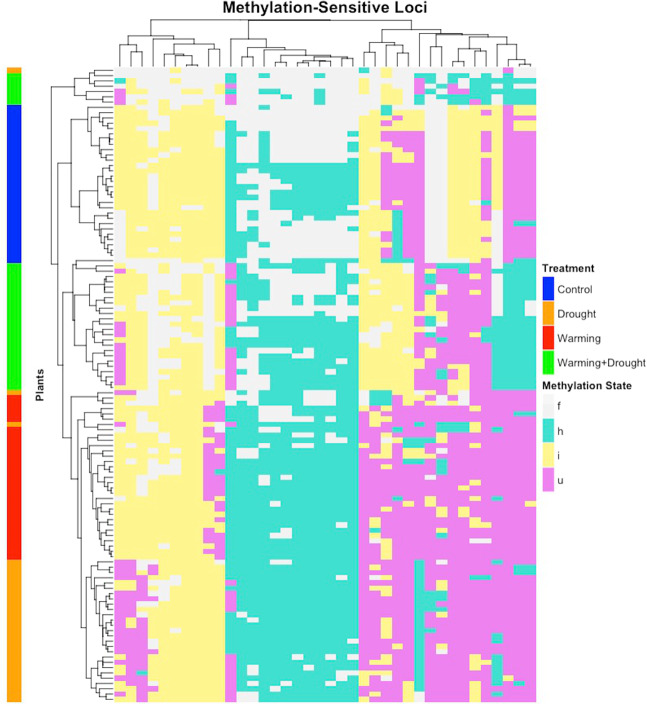
Heatmap of 38 MSL obtained after a *locus*-by-*locus Chi*-squared test (*p* value < 0.001 after Benjamini and Hochberg adjustment), showing differences in methylation patterns between climatic treatments. Methylation states are f: full methylation; h: hemimethylated, i: inner cytosine methylation, and u: unmethylated. Samples (rows) and *loci* (columns) were clustered using the average linkage method.

In addition, Fig. 4 shows a *locus*-by-*locus Chi-*squared test analysis, which allowed to detect a group of 38 *epiloci* (11% of the total) with strong differentiation among climatic treatments (*p* value < 0.001 adjusted by Benjamini and Hochberg multitest), since the 120 plants analysed were broadly grouped in four clusters corresponding to each one of the climatic treatments (Fig. 4, row clustering). Within this sub-*epiloci* group, the proportions of DNA methylation states were similar to those shown in Table 3 for multi-*locus* analysis. Those *epiloci* showing uninformative state in C and WD plants, which can be also considered as full methylated (f), were mainly hemimethylated (h) in D and W plants. Figure 4 also shows that some unmethylated *epiloci* (u) in treated plants (i.e., plants subjected to D, W and WD treatments) were internally methylated (i) in C plants. Moreover, some others unmethylated *epiloci* in C, D and W were found internally methylated or hemymethylated in WD plants (i/h).

### Temporal changes in plant species frequencies

**Fig. 5:**
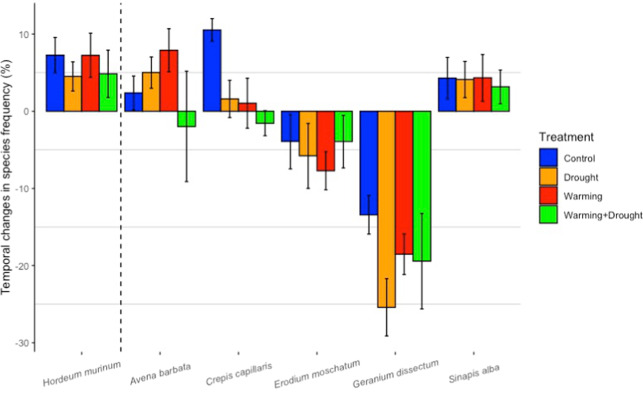
Changes in species frequency. Temporal variation in the relative frequency of the most dominant plant species when comparing plant communities in 2017 (first spring after experimental treatment onset) and 2018 (second spring).

The analysis of the variation in the frequency of hare barley relative to other co-dominant species indicated that 1 year after the onset of the experiment, the Geraniaceae *Erodium moschatum* and especially *Geranium dissectum* decreased in all treatments, particularly in those subjected to increased temperature and decreased rainfall. In contrast, hare barley, the grasses *Avena barbata* and *Crepis capillaris*, and the Brassicaceae *Sinapis alba* tended to increase in 2018 (Fig. 5). Of the three grasses, only hare barley increased in abundance in all treatments. In fact, our study species (together with *S. alba*) was the only dominant species that increased its frequency in response to the combined effect of increased temperature and drought (i.e., WD treatment).

## Discussion

In this field study we evaluated the phenotypic and molecular responses of hare barley to drier and warmer conditions predicted by Climate Change models for the study area (Lindner et al. 2010). Our results show that hare barley displayed not only a remarkable phenotypic plasticity but also a high epigenetic diversity in response to increased temperature, decreased rainfall and the combination of both. Global methylation seems to be widely generalised in the genome of many plant species (Suzuki et al. 2008), being particularly frequent in species with high presence of transposable elements such as hare barley (Jakob et al. 2004; Sharifi-Rigi et al. 2014), and large genomes such as *Zea mays* (Diez et al. 2014). Specifically, our study species was found to have the largest genome within the *Hordeum* genus (Jakob et al. 2004). The epigenetic variation that exhibited hare barley between treatments was higher than the genetic diversity quantified for this species in the study area, which is a common feature even in genetically diverse plant species (Herrera and Bazaga 2010; Lira-Medeiros et al. 2010; Richards et al. 2012). Even though genetic variation exists within plots, as suggested by the genetic analysis of C plants, the overall low genetic diversity found between plots was an expected result taking into account the low distances between plots and that the study species is characterised by anemochorous dispersal and cleistogamous pollination (Fotiou et al. 2007). However, uncertainty about the potential effects of climatic treatments in selection remains, as germination and seedling survival of specific genotypes might be also influenced by different conditions (Fernández-Pascual et al. 2013), so that the observed differences could be the result of short-term, rapid adaptation, instead of epigenetic modulation. Results from the present study suggest that epigenetic diversity might be fundamental in acclimation to changing environmental conditions, most likely by altering gene expression, which is in line with the results found in other studies (Bossdorf and Zhang 2011; Li et al. 2012; Gayacharan 2013; Liu et al. 2018). Moreover, knowledge about phenotypic variation in response to different climatic conditions can help to implement management plans aimed at attenuating the potential impact of climate change on Mediterranean *dehesas*, for example by adapting grazing management to changes in plant growth and phenology induced by climate, given the importance of these features on palatability (Hussain and Durrani 2009).

### Phenotypic responses to different climatic scenarios

Hare barley exhibited a large phenotypic plasticity in response to climatic conditions. Plants subjected to warmer conditions (in W and WD) flowered earlier, and those subjected to combined stress (WD) showed a higher investment in leaf area per unit of leaf mass (i.e., higher SLA) and produced heavier seeds. Thus, the functional performance of hare barley was more sensitive to warming, particularly in combination with reduced rainfall, than to rainfall reduction alone. The increase in SLA with warming is consistent with the effect of temperature in reducing leaf thickness (Poorter et al. 2009; Lamaoui et al. 2018), although this could also result from side effects of OTCs on air relative humidity affecting leaf area and cell wall thickness (Piikkia et al. 2008). Contrary to our initial expectation, rainfall reduction did not cancel out the positive effect of warming on SLA, and so plants subjected to increased temperature and decreased rainfall did not produce leaves with trait values more associated to a water conservation strategy. This increased biomass allocation to leaf area could potentially make plants more susceptible to drought (Bongers et al. 2017). However, given the importance of this attribute in whole-plant carbon gain and growth (e.g., Poorter et al. 2009), plants with high SLA could be also more competitive in the uptake of resources, not only light but also soil water and nutrients. The decline in relative abundance of other co-occurring species such as *G. dissectum* and *E. moschetum*, particularly when exposed to potentially more stressful conditions, suggests that they were more affected by these climatic conditions than hare barley. Therefore, hare barley could benefit from an increased availability of nutrients and water no longer used by more stress-sensitive species in response to the isolated and combined effects of warming and reduced rainfall. Similar indirect interactions between climatic changes and species performance have been discussed elsewhere (Ogaya et al. 2003; Seifan et al. 2010; Rodríguez-Calcerrada et al. 2013). In plant communities, species hierarchy and dominance patterns may vary depending on the species´ specific competitive abilities under stress (e.g., Matías et al. 2018), with stress occasionally favouring non-dominant or subordinate species (Mariotte et al. 2013).

The potentially higher competitive ability detected for hare barley under warmer and drier conditions might help us to explain the observed changes in its reproductive ecology in the WD treatment; plants subjected to the combined effects of decreased rainfall and increased temperature advanced their flowering and produced bigger seeds. The advanced flowering phenology with warming is in agreement with the broadly known trend previously reported by other studies (e.g., Whittington et al. 2015; Valencia et al. 2016; Moore and Lauenroth 2017). This early phenology likely allowed plants to produce bigger seeds, since early flowering plants could potentially allocate resources to reproduction over an extended period of seed maturation (Wolkovich and Cleland 2014). The production of large seeds has been interpreted as a successful regeneration strategy under stressful conditions, where competition among seedlings might arise due to high resource limitation (Moles and Westoby 2004; Muller-Landau 2010).

### Epigenetic responses to different climatic scenarios

Both the level and pattern of methylation in hare barley varied substantially among the climatic treatments, with the combination of D and W inducing a clearly different epigenetic response compared to that promoted by D and W when applied separately. In terms of global DNA methylation levels, plants growing under control conditions differed from those exposed to reduced rainfall (D) and increased air temperature (W). These results were corroborated by pairwise AMOVA of epigenetic diversity among treatments, which allowed us to discern that the highest differences in epigenetic variation appeared between WD plants and those exposed to single D and W stresses, and then between C plant and those exposed to singles stresses (W and D). Different studies have reported that the combination of biotic and/or abiotic stresses results in a unique response that is not the mere addition of the effects caused by each stress separately. In Kentucky bluegrass, for example, the simultaneous presence of heat and drought caused higher reductions in photosynthesis and leaf photochemical efficiency than drought or heat alone (Jiang and Huang 2000). In wheat, the synthesis of heat-shock proteins induced by combined heat and drought was higher than after the application of only one of the stresses separately (Grigorova et al. 2011). Transcriptomic analysis by DNA chips revealed that acclimation to combined heat and drought in *Arabidopsis thaliana* also resulted in a unique response, with genes that were neither induced nor repressed by drought or heat stress alone responding to the combination of both stresses (Rizhsky 2004). However, and despite epigenetics constitute an emerging discipline with increasing interest in plant adaptation studies, few works have focused on the epigenetic control of gene expression in response to combined stresses. The comparison of combined stresses at the same time has been mostly analysed at the post-transcriptomic level. For instance, Forestan et al. (2016), via RNA-seq analysis, identified long non-coding RNAs and small interfering RNA with specific roles in the epigenetic regulation of gene expression in maize in response to drought and salt stress. To the best of our knowledge, the only study experimentally testing the combined effects of heat and drought at the epigenetic level was carried out by Liu et al. (2015), in which the enrichment of Gene Ontology terms (i.e., terms representing gene products classified as cellular components, molecular functions or biological processes) was related to epigenetic regulation in a transcriptomic analysis conducted in *Triticum aestivum*.

In terms of methylation patterns, we also found strong differences among plants as a function of the climatic treatment. Plants subjected to decreased rainfall (i.e., D and WD treatments) exhibited the highest levels of the unmethylation state, while plants growing under C and W treatments showed the highest levels of the uninformative state. As mentioned before, this methylation state is considered as uninformative due to the putative presence of changes in the restriction targets. However, it might be also due to the full methylation of the target, which corresponds to a repression of the gene expression. Therefore, these results might imply higher hypomethylation and subsequent upregulation of putative responsive genes in those plants subjected to drier conditions. This explanation is supported by previous studies. For instance, drought-susceptible rice genotypes showed repression of drought-responsive genes via hypermethylation under drought conditions, while drought-tolerant genotypes exhibited hypomethylation and subsequent induction of gene expression (Gayacharan 2013). More recently, overall hypomethylation was detected in drought-tolerant genotypes of wheat, in contrast to genome-wide hypermethylated drought-susceptible genotypes (Kaur et al. 2018). Moreover, the *locus*-by-*locus* analysis performed in our study confirmed the observed frequencies of methylation states for 41 *epiloci* significantly related to the four climatic treatments. Thus, higher proportion of unmethylated *loci* were found for standalone treatments (D and W). In addition, the proportion of uninformative state (putatively associated to full methylation and subsequent gene repression) was higher in C plants when compared to treated plants (i.e., D, W and WD). These results seem to confirm the existence of demethylation processes behind the overexpression of stress-responsive genes associated to these 41 *epiloci*.

## Conclusions

The substantial differences in epigenetic diversity and the presence of specific epigenetic patterns found in response to each climatic treatment suggest that molecular responses underlying the reprogramming of gene expression and metabolic processes could be driving the functional stability of some dominant grass species (such as hare barley) under different environmental scenarios (Zhu 2016). The potential role of epigenetic regulation as a mechanism of adaptation to new environmental conditions is reinforced by the low genetic diversity that exhibited the study species when comparing the four climatic treatments.

These results will serve as foundation for further analysis on how epigenetic variation underlies acclimation to different climatic conditions in hare barley. Understanding how genome-wide methylation affects plant function and ultimately species frequencies will improve our predictions on how climate change might alter plant community composition and ecosystem processes under future environmental scenarios. Transgenerational plasticity (e.g., via changes in seed size), influenced by epigenetic regulation during adaptive responses of parental individuals to environmental stress, opens new exploratory venues of research. Further analysis such as bisulfite sequencing and transcriptomic analysis (Metzger and Schulte 2017) will allow correlating these *epiloci* to candidate genes with specific roles in phenotypic variation and functional responses to environmental changes.

## Supplementary information

Supplementary Data

## Data Availability

AFLP and MSAP fingerprints of hare barley control plants and subjected to experimental conditions, respectively, can be found at Zenodo repository under the following Digital Object Identifier: 10.5281/zenodo.3531668.
