# Supplementary material for: Epigenetic responses of hare barley (Hordeum murinum subsp. leporinum) to climate change: an experimental, trait-based approach
Source: Heredity (Edinb). 2021 Feb 19;126(5):748–62. doi: 10.1038/s41437-021-00415-y (PMC8102545; doi:10.1038/s41437-021-00415-y)
Supplement: Supplementary file 1 — Supplementary Data [file 41437_2021_415_MOESM1_ESM.pdf]

Heredity

*Heredity* **Supplementary Data**

**Epigenetic responses of hare barley (*Hordeum murinum* subsp. *leporinum*) to climate change: an experimental, trait-based approach.**

Authors: Víctor Chano, Tania Domínguez-Flores, Maria Dolores Hidalgo-Galvez, Jesús Rodríguez-Calcerrada, Ignacio Manuel Pérez-Ramos

The following Supplementary Data is available for this article:

**Figure S1:** Global distribution map of *Hordeum murinum* L.

**Figure S2:** Experimental setup

**Figure S3:** Soil surface air temperature and soil volumetric water content

**Table S1:** Adapters and primers used for AFLP and MSAP protocols

**Table S2:** Interpretation of banding patterns obtained with the MSAP technique

**Table S3:** Statistical analysis results for significant phenotypic traits

**Table S4:** Results from pairwise analyses of molecular variance (AMOVA) between pairs of experimental plots

**Figure S1.** Global distribution map of *Hordeum murinum* L. based on Kalwij *et al.* (2014; DOI: 10.1371/journal.pone.0085306).

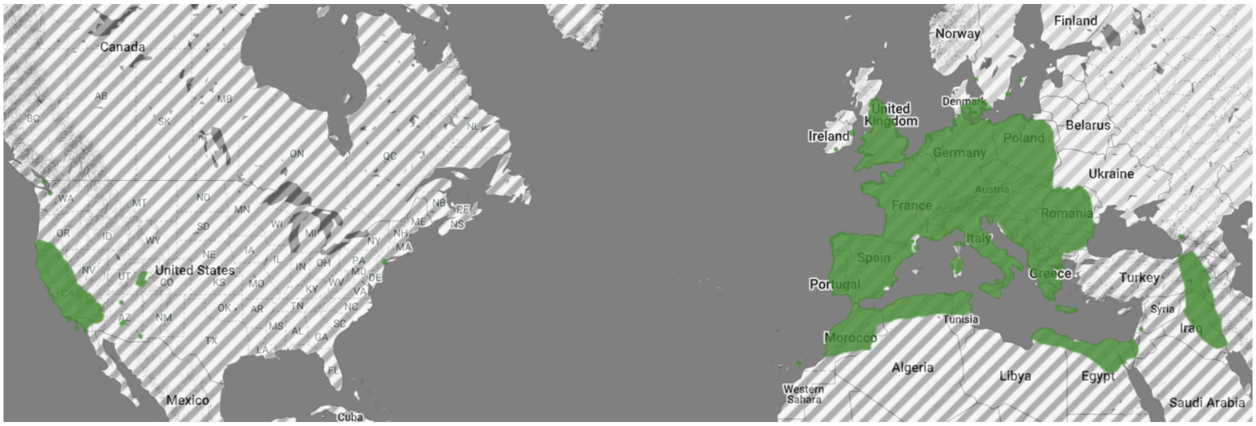

**Figure S2.** Experimental setup. Picture showing rainout shelter for dryer conditions (Drought and Warming+Drought) and open top chambers (OTC) for warmer conditions (Warming and Warming+Drought).

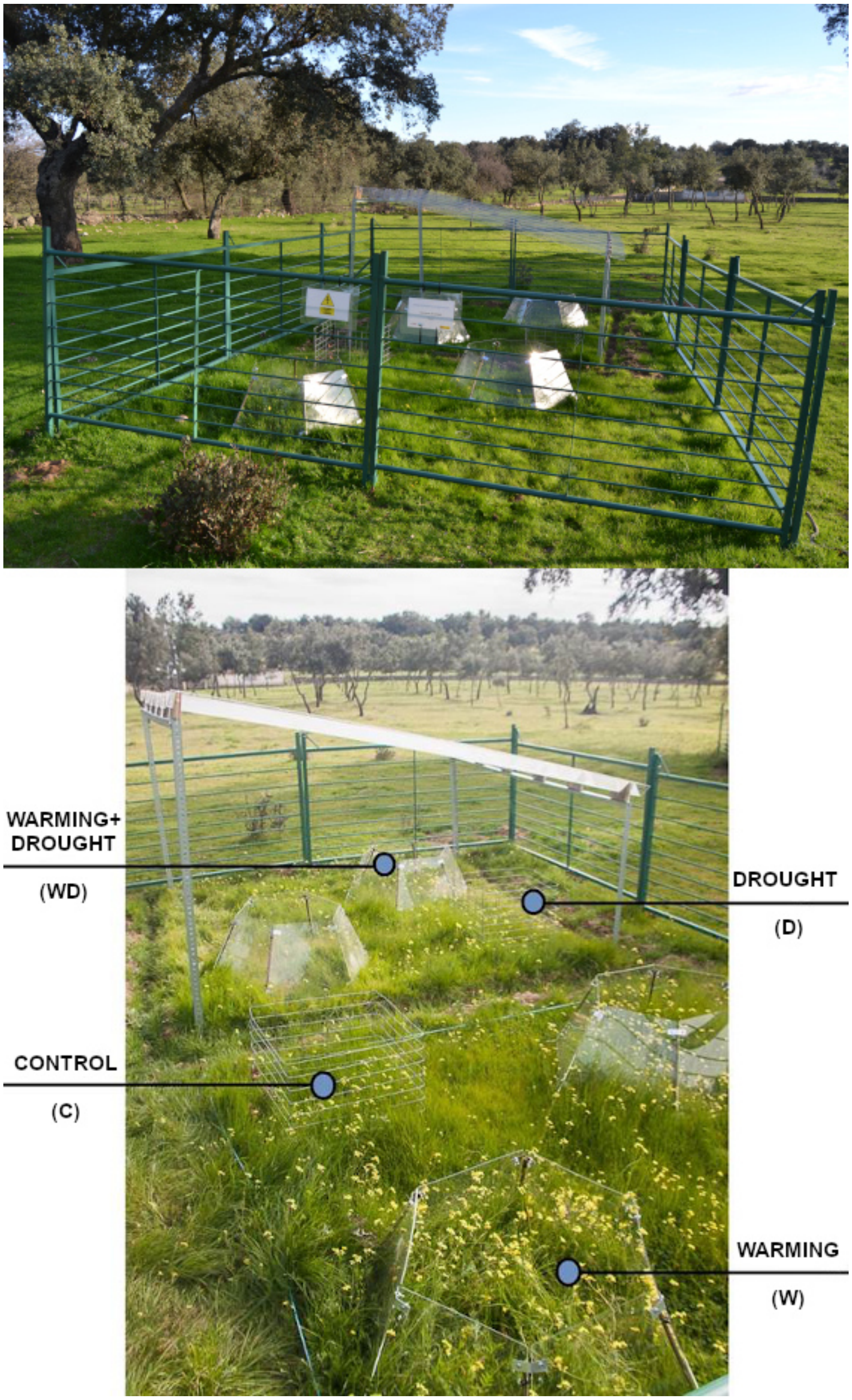

**Figure S3.** Soil surface air temperature and soil volumetric water content in the four climatic treatments during the ongoing experimental process. A. Monthly variation in air temperature (ºC). B. Variation in volumetric soil water content (%).

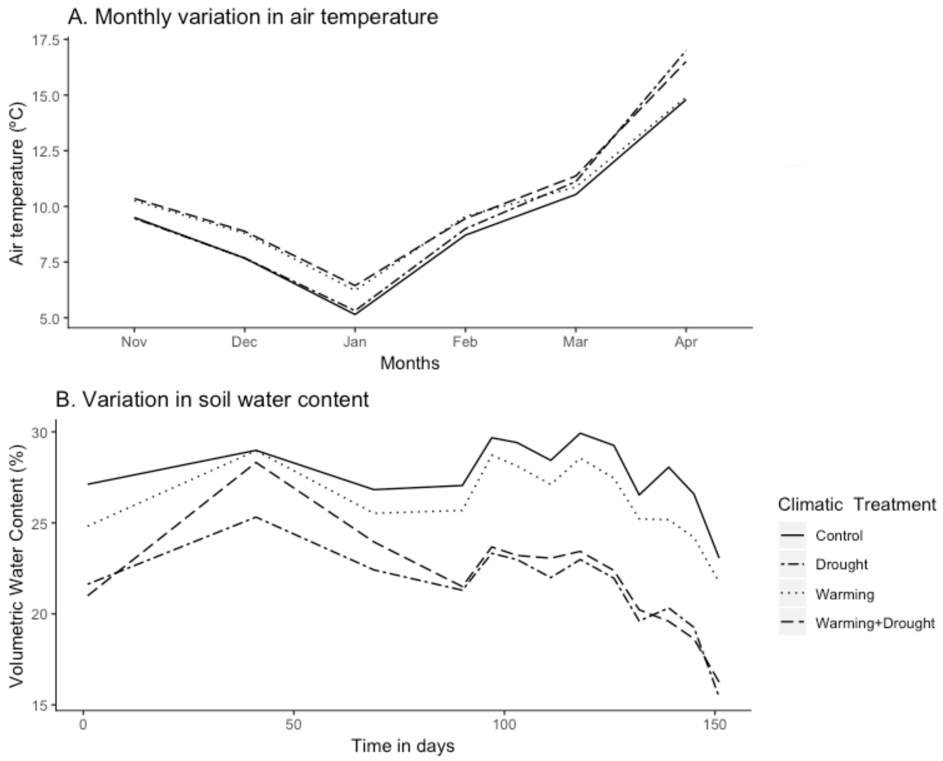

**Table S1.** List of oligonucleotide sequences used for adapters construction and pre-selective and selective primers.

| **Primer name** | **Sequence (5'→3')** |
|---|---|
| *Adapters* | |
| *EcoRI*-Adapt1 | CTCGTAGACTGCGTACC |
| *EcoRI*-Adapt2 | AATTGGTACGCAGTC |
| *MseI*-Adapt1 | GACGATGAGTCCTGAG |
| *MseI*-Adapt2 | TACTCAGGACTCAT |
| *HpaII/MspI*-Adapt1 | GACGATGAGTCTCGAT |
| *HpaII/MspI*-Adapt2 | CGATCGAGACTCAT |
| *Pre-selective primers* | |
| E01 | GAC TGC GTA CCA ATT C a |
| M02 | ATG AGT CCT GAG TAA c |
| H01 | GAT GAG TCT CGA TCG G a |
| *Selective primers* | |
| E32* | GAC TGC GTA CCA ATT C aac |
| E33* | GAC TGC GTA CCA ATT C aag |
| E34* | GAC TGC GTA CCA ATT C aat |
| E35* | GAC TGC GTA CCA ATT C aca |
| M53 | GAT GAG TCC TGA GTA A ccg |
| M56 | GAT GAG TCC TGA GTA A cgc |
| H31 | GAT GAG TCT CGA TCG G atc |
| H32 | GAT GAG TCT CGA TCG G act |
| H33 | GAT GAG TCT CGA TCG G aat |

Nucleotides in lowercase letters represent selective nucleotides for AFLP and MSAP analyses.
* Labelled with IRDye700 and IRDye800.

**Table S2.** Interpretation of methylation states of cytosines in the target sequence of the isoschyzomeric restriction enzymes HpaII and MspI according to the different banding patterns (adapted from Guevara et al., 2017; DOI 10.1007/978-1-4899-7708-3_9).

| | | Interpretation | Binary coding of band pattern (EcoRI/HpaII) / (EcoRI/MspI) |
|---|---|---|---|
| Status I | $^{5'}$CCGG<br>GGCC$_{5'}$ | Unmethylation | 1//1 |
| Status II | $^{5'}$C$^{m}$CG G<br>G GC$_{m}$C$_{5'}$ | Methylation of one or both inner cytosines | 0//1 |
| | $^{5'}$C$^{m}$CGG<br>G GCC$_{5'}$ | | |
| Status III | $^{5'm}$CCGG<br>GGCC$_{5'}$ | Hemymethylation of external cytosine | 1//0 |
| Status IV | $^{5'm}$C$^{m}$CG G<br>G GC$_{m}$C$_{m5'}$ | Full methylation/Uninformtive | 0//0 |

**Table S3.** Results from the Kruskal-Wallis/Welch's ANOVA test and Dunn-Bonferroni/Games-Howell post-hoc analysis of the influence of climatic treatments on some key phenotypic traits. H: Kruskal-Wallis statistic; d.f.: degrees of freedom; F: Welch's ANOVA statistic.

| ***Phenotypic trait*** | ***Kruskal-Wallis test*** | | | ***Benjamini-Hochberg test*** | |
|---|---|---|---|---|---|
| | ***H*** | ***d.f.*** | ***p-value*** | ***Pairwise-comparison*** | ***p-value*** |
| *Leaf Size* | 8.2948 | 3 | 0.0403** | C – D | 0.094* |
| | | | | C – W | 0.833 |
| | | | | C – WD | 0.094* |
| | | | | D – W | 0.107 |
| | | | | D – WD | 0.929 |
| | | | | W – WD | 0.107 |
| *Flowering Onset* | 8.8393 | 3 | 0.0315** | C – D | 0.363 |
| | | | | C – W | 0.041** |
| | | | | C – WD | 0.041** |
| | | | | D – W | 0.228 |
| | | | | D – WD | 0.228 |
| | | | | W – WD | 0.891 |
| *Seed Mass* | 10.598 | 3 | 0.0141** | C – D | 0.095* |
| | | | | C – W | 0.341 |
| | | | | C – WD | 0.341 |
| | | | | D – W | 0.392 |
| | | | | D – WD | 0.014** |
| | | | | W – WD | 0.085* |
| ***Phenotypic trait*** | ***Welch's ANOVA test*** | | | ***Game-Howell test*** | |
| | ***F*** | ***d.f.*** | ***p-value*** | ***Pairwise-comparison*** | ***p-value*** |
| *Specific Leaf Area* | 10.673 | 3 | 0.0001** | C – D | 0.048** |
| | | | | C – W | 0.135 |
| | | | | C – WD | <0.0001*** |
| | | | | D – W | 0.730 |
| | | | | D – WD | 0.0062*** |
| | | | | W – WD | 0.612 |

*** *p-value* < 0.01; ** *p-value* < 0.05; * *p-value* < 0.1

**Table S4.** Results from pairwise analyses of molecular variance (AMOVA) between pairs of experimental plots. Values correspond to *Phi*-statistic based on AFLP *loci*.

| | PLOT 1 | PLOT 2 | PLOT 3 | PLOT 4 | PLOT 5 | PLOT 6 |
|---|---|---|---|---|---|---|
| PLOT 1 | - | | | | | |
| PLOT 2 | 0.000$^{ns}$ | - | | | | |
| PLOT 3 | 0.036$^{ns}$ | 0.018$^{ns}$ | - | | | |
| PLOT 4 | 0.063$^{ns}$ | 0.069$^{ns}$ | 0.146$^{ns}$ | - | | |
| PLOT 5 | 0.066$^{ns}$ | 0.013$^{ns}$ | 0.000$^{ns}$ | 0.010$^{ns}$ | - | |
| PLOT 6 | 0.049$^{ns}$ | 0.000$^{ns}$ | 0.000$^{ns}$ | 0.091$^{ns}$ | 0.000$^{ns}$ | - |

ns*:* not significant.
